# Treatment of Post-Inflammatory Hyperpigmentation in Skin of Colour: A Systematic Review

**DOI:** 10.1177/12034754241265716

**Published:** 2024-07-29

**Authors:** Kristie Mar, Bushra Khalid, Mahan Maazi, Rayan Ahmed, Ou Jia (Emilie) Wang, Touraj Khosravi-Hafshejani

**Affiliations:** 1 Faculty of Medicine, University of British Columbia, Vancouver, BC, Canada; 2 Department of Dermatology and Skin Science, University of British Columbia, Vancouver, BC, Canada

**Keywords:** PIH, laser, retinoid, SOC, FST, hydroquinone

## Abstract

Post inflammatory hyperpigmentation (PIH) affects all skin types with a heightened predilection for darker skin tones. Its course is chronic once developed and treatment is often difficult. This systematic review aims to summarize the treatment outcomes for PIH with a focus on skin of colour (SOC) individuals. A literature search was conducted using MEDLINE (from 1946), Embase (from 1974), PubMed, and Cochrane in adherence to the Preferred Reporting Items for Systematic Reviews and Meta-Analysis guideline. Results from 48 studies summarized 1356 SOC individuals. The mean age was 29 years (n = 1036) and 78% were female (n = 786). The ethnic prevalence was 70% Black, 27% Asian, and 3% Latin. Overall, 20% were Fitzpatrick skin type (FST) III, 40% FST IV, 34% FST V, and 6% FST VI. Most cases were precipitated by inflammatory conditions (89%) and localized to the face (83%). The most frequently reported interventions were topical retinoids (22%) and laser therapy (17%). Partial improvement was seen in 85% and 66% of participants, respectively. Laser was the only intervention that offered complete resolution in a subgroup of patients (26%); however, there were reported cases of PIH exacerbation following treatment. Chemical peels (9%) and hydroquinone (7%) were among other treatments with less effective outcomes. PIH and its persistence is a prevalent issue, significantly affecting many affected individuals with darker skin tones. Our results show a lack of robust efficacy across all treatment modalities. There is considerable room for improvement in interventions for at-risk populations.

## Introduction

Post-inflammatory hyperpigmentation (PIH) is a form of acquired hypermelanosis often observed as a consequence of endogenous factors, such as inflammatory conditions, or exogenous factors, such as mechanical trauma.^1-4^ PIH is characterized by an excess production or deposition of melanin in the epidermis and/or dermis, resulting from inflammation.^1-3^ The darkening of the skin is attributed to the activation of melanocytes by inflammatory mediators.^1,2^ It is frequently exacerbated by ultraviolet radiation and excess sun exposure.^1,4^ PIH tends to be more prominent and enduring in individuals with darker skin tones, particularly in skin of colour (SOC) individuals [ie, Fitzpatrick skin type (FST) III-VI], who have an increased size of melanosomes, quantity of melanin, as well as increased eumelanin.^3,5,6^ While the prevalence of PIH varies globally, it ranges from approximately 0.4% to 10.0% in African Americans.^1,6,7^

PIH results in significant psychosocial distress and a diminished quality of life for affected individuals.^1,8^ However, women with SOC exhibit a particularly high concern for clearing PIH.^7^ Despite its significant impact, research on treatments for PIH has traditionally centred around individuals with lighter skin tones, leading to noticeable disparities in care. The available treatment options are limited, and there is a lack of efficacy data applicable in SOC.^1^ Common therapeutic approaches involve the use of topical treatments, for example, retinoids, tranexamic acid (TXA), and hydroquinone.^3,7^ Further investigation into light and laser therapy has also been conducted.^3^

Previous reviews have investigated topical treatments and energy-based devices for PIH among the general population.^3,5^ In 2010, a literature review was conducted on clinical features and treatment options particularly for SOC patients.^6^ However, an updated review on outcomes for darker skin tones is currently lacking. The primary objective of this systemic review is to summarize the treatments and their outcomes for PIH, with a focus on individuals with SOC. The overarching goal is to equip physicians with inclusive insights to enhance patient outcomes across all skin tones.

## Methods

This systematic review was conducted with adherence to the Preferred Reporting Items for Systematic Reviews and Meta-Analyses extension for Scoping Reviews (PRISMA-ScR).^9^ The study protocol was registered with the PROSPERO database (CRD42022366067).

### Eligibility Criteria

Eligibility criteria for this review were established as follows:

- *Population*: Individuals of any age and sex with SOC (non-Caucasian, FST III-VI) diagnosed with PIH undergoing intervention, regardless of disease severity, treatment received, or comorbid conditions.
- *Intervention*: Any intervention aimed at addressing existing PIH was included.
- *Comparator*: Untreated SOC individuals with PIH, or in the absence of a comparator, open label studies where outcomes were assessed in treated SOC individuals with PIH.
- *Outcomes*: Patient age/sex, FST III-VI, racial/ethnic patient representation, PIH duration, treatment, treatment outcomes focused to race/ethnicity/FST, scoring indexes, recurrence, time to resolution, follow-up, adverse effects.
- *Study Design*: Eligible studies included randomized controlled trials (RCTs), cohort (prospective and retrospective) studies, cross-sectional studies, case-control studies, case-series, and case reports.

### Search Strategy

A literature search was conducted using MEDLINE (from 1946), Embase (from 1974), Cochrane Database of Systematic Reviews, and PubMed on July 2, 2023, using the OVID interface. No restrictions were applied. Title, abstract, and full-text screening was conducted independently by 3 reviewers (KM, BK, and MM) using Covidence online systematic review software (www.covidence.org).

### Data Extraction

Data extraction was completed by 3 reviewers (KM, BK, and MM) on a standardized extraction form. Extracted data included publication year, country, study design, number of participants, age and sex of participants, duration of PIH, race/ethnicity, FST, treatment regimen received, outcomes, scoring indexes, healing time, and adverse effects.

### Data Synthesis

After data collection, it was determined that the lack of standardized reporting necessitated a pooled and separate reporting of results. For studies with certain variables, such as, scoring indexes, adverse effects, outcome significance, and patient satisfaction that could not be pooled, a narrative synthesis was completed. For pooled intervention results, outcomes were reported as complete, partial, or no response.

### Definitions

- *Complete response (CR)*: complete resolution as defined by the criteria outlined in each study. For example, one study reported “one case of complete PIH resolution.”
- *Partial response (PR)*: partial resolution in size or pigment level defined by the criteria outlined in each study. For example, one study used the post acne hyperpigmentation index (PAHPI) to report a significant decrease in the score from pre- to posttreatment (9.67-5.47, P = .034).
- *No response (NR)*: undetectable change defined by criteria outlined in each study. For example, one study used the Global Assessment Scale (GAS) to report that 18% remained “unaltered.”

## Results

### Patient Characteristics

After title and abstract screening of 3205 articles and full-text review of 261 articles, 46 studies were ultimately included in this review, summarizing 1356 SOC patients (Supplemental Figure 1). Most of the studies came from the United States (31%), Korea (11%), China (9%), and the United Kingdom (9%; Supplemental Table 1). Study types included 31% case reports, 30% experimental studies, and 27% RCTs.

For SOC individuals, the mean age was 29 years (n = 1036) and 78% were female (n = 786; Supplemental Table 2). The ethnicity/race distribution was reported in 849 participants as: Black (70%), Asian (27%), Hispanic/Latin (3%), and other (0.2%). There were no reported cases among Middle Eastern/Arab, Pacific Islander, or Indigenous categories. Of 570 cases with available data on FST, FST III accounted for 20%, FST IV for 40%, FST V for 34%, and FST VI for 6%.

### PIH Characteristics

The average duration across the studies for PIH prior to treatment was reported as 21 months (n = 167). Among 1332 cases, the lesions were most frequently located on the face (92%), followed by the axillae (4%) and extremities (3%). Less common sites are further described in Supplemental Table 2. Among 83 reported cases, 48% were categorized as localized, 45% diffuse, and 8% symmetrical.

### Precipitating Factors

Precipitating factors contributing to the development of PIH were reported in 1225 cases. Inflammatory conditions accounted for 89% of the total cases (n = 1089), with acne being the predominant cause (97%, n = 1060; Supplemental Table 2). Trauma-related PIH constituted 11% of the cases (n = 134), predominantly caused by laser therapy (27%, n = 36), followed by hair removal techniques (26%, n = 35), light therapy (23%), and chemical peels (20%). Drug-induced PIH comprised 0.2% of the cases, with 2 unidentified drug eruptions.

### Pooled Intervention Results

#### Treatments and outcomes

Among 346 (26%) participants who did not receive treatment, none had a CR, 62% achieved partial pigment reduction, 4% achieved partial size reduction, and 33% had NR. There was an average time to resolution of 68 days (n = 55) and follow-up time of 3 months (n = 130; Supplemental Table 3).

The primary treatment approach utilized a single topical agent (34%), with topical retinoids being the most frequently reported treatment modality. Subsequently, device-based treatments, predominantly laser therapy, constituted 25% of reported cases. Alternative regimens have also been explored, yielding variable outcomes.

Among all therapeutic approaches, topical retinoids were the most investigated, used by 294 (22%) participants. On average, the product was applied 119 times. Although no participants had CR of PIH, 64% out of 118 patients exhibited partial pigmentation reduction, 21% had partial size reduction, and 14% had NR to treatment. The pooled mean follow-up time was 4 months (n = 143). Laser therapy was completed on 227 (17%) participants with an average of 4 sessions. Among 165 patients, 26% had CR, 66% had partial pigment reduction, and 33% exhibited NR. There was a mean resolution time of 140 days (n = 22) and a follow-up time of 8 months (n = 141). Chemical peels, used by 123 participants (9%) and applied an average of 5 times, produced partial pigment reduction in 67% (n = 40) and NR in 33% (n = 20) of cases. The mean resolution time was 28 days (n = 8) with a follow-up time of 4 months (n = 70). Other various modalities with smaller sample sizes were investigated, for example, intense pulsed light (IPL) therapy, Bakuchiol, tyrosinase inhibitor isobutylamido-thiazolyl-resorcinol (Thiamidol), topical niacinamide, compounded hydroquinone creams, and high-intensity focused ultrasound (HIFU; Supplemental Table 3 and Supplemental Figure 2).

### Analysis of Select Intervention Results

#### Therapeutic topical intervention

##### Topical retinoids

Tretinoin, adapalene, and tazarotene all showed significant partial improvements in PIH after 12 weeks. Three studies found significant partial efficacy of topical tretinoin as a monotherapy at a concentration of either 0.04%, 0.05%, or 0.1% among 258 patients.^10-12^ One study used the Post-Inflammatory Hyperpigmentation Severity Scale (PIHSS) to measure a range of improvement from 77% to 100%.^11^ Another study reported a decrease in number and density of macules using 0.1% adapalene gel among two-thirds of their patients.^13^ A third study reported that 0.1% tazarotene resulted in an average reduction in pigmentary intensity by an approximate 15% decrease or 1.2 grades.^14^ Side effects were reported for tretinoin and tazarotene including mild to moderate local irritation, erythema, desquamation, and burning and retinoid dermatitis with tretinoin.^10,11,14^

##### Other topical monotherapies

Two studies found that topical steroids alone could partially improve PIH in 17 patients.^15,16^ An RCT evaluated 0.05% desonide and found a significant mean reduction of 28% in lightness index values.^16^

One study observed significant improvement in trichloroacetic acid-induced PIH with bakuchiol measured with a 10% score reduction of the Investigators Global Assessment (IGA).^17^ However, no significant improvement was noted for acne-induced PIH. Furthermore, a significant negative correlation was observed between the degree of PIH and the duration of time elapsed since treatment initiation.

Last, a single case report of cysteamine cream showed a significant reduction of the melanin index (MI) score by approximately 9%.^18^

##### Combination therapy

Both compounded hydroquinone formulations at 2% and 4% demonstrated noteworthy improvements in PIH, with no distinct advantage for one combinatorial regimen emerging over others. When paired with other treatments, more significant improvement was reported with chemical peels compared to laser therapy.

One study used the Hyperpigmentation Activity and Severity Index (HASI) to evaluate the efficacy of a modified Kligman’s formula (MKF) versus hydroquinone combined with a glycolic acid (GA) peel.^19^ After 12 weeks, the GA peel group reported a significant 36% reduction in the HASI score, while the MKF-alone group reported a significant decrease of 24%. Another study compared 3 arms: hydroquinone 4%, tretinoin 0.05%, fluocinolone acetonide 0.01% and laser, versus laser alone versus topical agents alone.^20^ Despite some partial improvement in all groups, there was no significant difference between them. In terms of side effects, one person had worsened PIH following laser treatment.

One study observed that combined topical clindamycin and tretinoin resulted in a consistent reduction of PIH by 1.2 grades or 15% reduction of the PIHSS. Most patients had minimal adverse effects of scaling, pruritus, and burning.^21^

One study reported significant partial improvement with Thiamidol and sunscreen, with an approximate 8% decrease in MI score and 25% patient self-reported improvement.^22^

#### Therapeutic device intervention

##### Laser therapy

Twelve studies evaluated the use of different lasers as a monotherapy for PIH in SOC patients. Laser types included 1064 nm QS Nd:YAG laser, 755 nm alexandrite picosecond laser, 1927 nm thulium fiber laser, fractional carbon dioxide (CO_2_) laser, 750-picosecond pulse using a 755 nm Alexandrite laser, and QS ruby laser. Nearly every laser type investigated, except for the QS ruby laser, exhibited noteworthy decreases in PIH. None reported an exacerbation of PIH as an adverse effect; however, a case was reported under combination therapy and with the QS ruby laser.

Five studies evaluating 1064 nm QS Nd:YAG laser treatment in a total of 110 patients, with a pooled mean of 5.6 sessions, reported significant reduction in PIH across all participants, with one case of complete PIH resolution.^23-27^ One study reported their treatment response using a 5-point grading scale: excellent (75%-100%) in 20% of patients, good (50%-74%) response in 75% of patients, and fair (25%-49%) response in 5% of patients.^28^

One study reported no improvement at all with the QS ruby laser with concerns of hyperpigmentation/hypopigmentation as an adverse effect, surpassing the pretreatment appearance.^29^

Three studies investigating the efficacy of a 755 nm alexandrite picosecond laser, with a pooled mean of 4 sessions, found moderate resolution in 2 patients, and complete resolution in one.^30-32^ One patient reported significant improvement (50%-75%) assessed with the VISIA (VISIA^®^ Skin Analysis System) score.^30^

Two studies demonstrated significant improvement, rated by 2 dermatologists using before and after photography (43% ± 25% improvement), with the 1927 nm laser in 61 patients over the course of 2 to 5 sessions. No significant differences were found with different numbers of treatments or with different FST.^33^

##### Intense Pulsed Light

Two studies found moderate efficacy and tolerability with IPL therapy, ranging from 3 to 8 sessions, in 85 Asian patients of FST III-V. A 5-point grading scale revealed a greater than 50% reduction in PIH in 13 patients after 2 months of treatment. In addition, the GAS revealed that 35% of 60 patients were “very improved,” 43% had “improved,” and 18% remained “unaltered.”^34^ Adverse effects included transient erythema, dryness, and pruritus at treatment sites, which resolved within 48 hours in most patients.^34,35^

##### High-intensity focused ultrasound

One study found greater efficacy for HIFU to treat Ultraviolet B-induced hyperpigmentation in FST IV compared to FST III, but overall had insignificant results with treatment in general.^36^ Erythema, edema, and skin tenderness occurred in all 20 participants. Ecchymoses were observed in 1 patient.

#### Therapeutic chemical peel

Nine studies documented the partial effectiveness of superficial peels including salicylic acid (SA), GA, phytic acid (PA), and lactic acid (LA) in reducing PIH lesions.

Two studies reported improvement with unclear significance using SA peels at a concentration of 20% to 30% in a total of 11 patients.^37,38^ One study measured significant difference on the treatment side versus control side with Visual Analog Scale score difference of approximately 32%. However, comparisons by raters were not significant. When surveyed, most patients perceived the treatment as effective. After 2 weeks of treatment, adverse effects reported were: burning (30%), redness (40%), dryness (10%), crusting (40%), itching (70%), hyperpigmentation (40%), and hypopigmentation (10%).^37^

Two studies reported on the significant partial efficacy and tolerability of GA peels when combined with other active ingredients. Formulas investigated were serial GA peels combined with MKF^19^ and serial GA peel, on 25 patients.^39^ The latter reported a HASI score decrease by 50% after 22 weeks.^39^ Adverse events recorded included mild erythema, superficial desquamation, and superficial vesiculation.^39^

One study showed comparative efficacy of LA, incorporated into Jessner’s peel, when compared with SA (30%).^40^ They reported a 31% and 25% reduction of PAHPI score in the LA and SA groups, respectively. One patient reported prolonged erythema and PIH. Another study reported significant partial improvement with LA incorporated with SA and other ingredients, measured with the IGA.^41^

The final study reported on the significant partial efficacy of a PA peel in 15 patients measured with a significant 26% decline in PAHPI score in the PAHPI.^42^

#### Therapeutic systemic intervention

One study observed near-complete resolution with oral isotretinoin for 4 months.^43^ No adverse events were recorded.

## Discussion

Treating PIH poses challenges and can potentially worsen the condition.^6,33,44^ Limited research exists on the safety and effectiveness of interventions for SOC individuals. Among available studies, there is a lack of robust efficacy across all treatment modalities. The only treatments with notable success over no treatment were Thiamidol, laser therapy, and topical retinoids.

Our study observed a higher prevalence of females experiencing PIH, particularly on the face. This predominance could be attributed to women’s heightened motivation to address PIH due to gender-biased social scrutiny of appearance.^45^ Perhaps the intersection of being female and having SOC contributes to an overall compounded impact on quality of life.^45,46^ However, it is crucial to emphasize that PIH affects individuals of both sexes, all skin tones and ethnicities, and other bodily locations as well.

Historically, dermatologic research has lacked documentation of race/ethnicity.^47,48^ Much of the earlier research focused on Caucasian individuals, inferred from recorded FST, or included images.^47,48^ In this study, the predominant FST was type IV and V. The predominant race/ethnicity identified was Black, followed by Asian and Hispanic individuals, consistent with existing literature.^7^

Dark-skinned individuals exhibit a heightened predisposition to PIH, attributed to molecular, cellular, and structural factors.^49^ Skin pigmentation level has been linked to melanocyte size, with larger cells in dark skin transferring more melanosomes to the epidermis due to heightened tyrosinase activity and larger, more acidic dendrites compared to light skin.^4,50,51^ Investigators have also found structural differences in the epidermis and dermal-epidermal junction, increased oxidative stress, impaired cutaneous vascular function, and elevated inflammatory markers like interleukin-6 and C-reactive protein in African Americans.^4^ Furthermore, dark-skinned individuals are less likely to apply photoprotection.^4^

A review reported months to years for PIH resolution, with the possibility of permanance.^6^ Our results also demonstrated prolonged duration and may be indicative of limited treatment options or increased difficulty in clearance. Furthermore, causes of PIH varied, with acne being the predominant inflammatory condition and laser being the predominant external cause. The exact mechanism of laser-induced hyperpigmentation is not fully understood but is likely exacerbated by heat production in the skin.^44^

Without treatment, our analysis revealed that zero SOC individuals were able to completely clear their PIH and a significant portion had no improvement at all. However, nearly two-thirds had partial resolution, raising the question of whether an improvement of PIH during a treatment can be partly attributed to spontaneous improvement over time rather than the inherent efficacy of the medication. Nevertheless, one cannot discount that most of these patients have had established PIH for over a year. Perhaps the pivotal consideration is quantifying the incremental benefit conferred by treatment. In the absence of treatment, strict photoprotection as prophylaxis is important.^3,47^

Of those treated, topical retinoids demonstrated a superior efficacy in size reduction of PIH compared to other topical regimens and demonstrated pigment reduction in most SOC patients. Although the improvement seen is slightly less pronounced compared to results in non-SOC individuals,^3^ they remain a viable treatment option offering comparable efficacy in both SOC and Caucasian patients.^52^ The overall tolerability has been found to be greater in Black patients than in White patients,^52,53^ and may contribute to better adherence. Caution is advised regarding retinoid dermatitis, which may potentially exacerbate PIH in dark skin.^7,54^ Risk mitigation could include starting at lower dosages and slowly increasing the concentration of the retinoid.^7,54^

Research on combination therapies in SOC is still in its nascence, particularly on combinations with topical retinol and hydroquinone. While hydroquinone combined with a retinoid or laser showed partial improvement, consistent with the literature,^3^ its combination with steroids yielded suboptimal results. Some investigators suggest that combining treatments can mitigate adverse effects of hydroquinone therapy,^6,55^ but our analysis did not reveal any significant difference in adverse effects between hydroquinone with or without steroids. Other researchers suggest that hydroquinone combined with additional ingredients (eg, retinoids or GA) can exhibit improved efficacy; however, this aspect remains an underexplored area of research.^6,55^ Other investigators suggested that topical hydroquinone should be a first-line treatment for SOC^6^; nevertheless, our analysis did not reveal any superiority over alternatives.

Although Thiamidol, a potent tyrosinase inhibitor,^22^ showed improvement, its combination with sunscreen raises the question of which ingredient contributed more significantly to the success observed in the study. The effect of sunscreen in prevention of PIH in SOC has been documented in the literature.^56,57^ Whether it offers the same benefits for treating established PIH remains unanswered.

Bakuchiol, an antimicrobial compound with anti-inflammatory and antioxidant properties, has gained popularity for improving the appearance of acne lesions and melasma,^17,58^ leading to speculation that it could also aid in PIH.^58^ Although Lyons et al^17^ observed mixed results, it may be attributed to the significant variation seen in baseline PIH among a small sample size. A longer follow-up duration might have revealed more clinically measurable improvements and further research is warranted.^17^

Other treatments explored, such as topical cysteamine and systemic retinoid, necessitate further investigation with larger sample sizes to establish their efficacy. In addition, topical agents such as vitamin C derivatives, azelaic acid, and cosmeceuticals have been considered as therapeutic options,^3^ but there are no available studies with specific results for SOC patients with PIH. Similarly, certain preventative measures for PIH on SOC, for example TXA and sunscreen, have not been evaluated as an intervention after PIH has developed, and the corollary is also true for intervention agents.^17,58^

Among all treatments explored for individuals with SOC, laser therapy stands out as the only option that demonstrated the ability to completely clear PIH. While the QS ruby laser yielded poor results, various studies have documented significant success with others. Previous literature has indicated that lasers with longer wavelengths, such as 1064 nm, are considered safer for darker skin tones.^44^ This is attributed to the more efficient absorption of energy by dark skin.^44^ Moreover, picosecond lasers, known for emitting short pulses, are also recognized as safe for darker skin tones due to their ability to minimize unwanted heat damage.^44,59^ These lasers enable deeper penetration into tissues and reduce absorption by epidermal melanin.^44^

Although the effects could not be stratified according to FST, Bae et al^33^ found no significant differences in the response from laser among individuals with types IV to VI. However, laser poses an increased rate of inducing PIH with repeated procedures (11%-17%), especially in darker skin types.^49,60-63^ Despite its efficacy, lasers remain a second-line option compared to topical agents due to cost considerations and the associated risk of complications.^44^

Our analysis found similar or potentially improved outcomes in SOC individuals undergoing chemical peels compared to the general population,^3^ with no observed recurrences. However, chemical peels exhibited increased adverse effects, specifically with SA, in comparison to alternative treatments. In addition, SA did not show significant reductions in PIH, especially when compared to GA. Speculation as to why GA may be a superior agent could be due to its structural similarity to ascorbic acid, resulting in a direct pigment reduction effect on the skin.^64^

SOC individuals have a well-documented elevated risk of developing PIH transiently after peels compared to the general population.^52,54^ Consequently, chemical peels should not be routinely recommended as a first-line treatment in this demographic but may have merit in refractory cases.^52,54^ Risk mitigation strategies can include pre- or posttreatment regimens, starting with lower concentrations, and emphasizing sun protection.^6,52,54^

This study has several limitations. First, the absence of a standardized scale for assessing PIH, coupled with instances where no scale is utilized, and unstandardized approaches to photography poses a challenge for consistent evaluation. To mitigate these complexities, a mixed methodology was employed to capture the outcomes to the best of our ability. Studies that included both light- and dark-skinned individuals but failed to stratify their outcomes were excluded. The results cannot be specified to each individual FST for similar reasons. Historically, many studies failed to report race/ethnicity or FST, resulting in our inability to capture non-SOC individuals for a direct comparative analysis. Additional limitations include small sample sizes and inconsistency in reporting variables. Last, despite this focused review, it is crucial to acknowledge the ongoing lack of representation of all ethnicities in the available literature.

## Conclusion

Despite a disproportionate effect of PIH on SOC individuals, there is a paucity of research on treatment efficacy. Our study highlights a critical need for the development of a universal PIH scale. While most treatments exhibited limited efficacy, laser therapy emerged as one of the few modalities capable of completely clearing PIH in select SOC individuals, albeit with associated high costs and risks of adverse effects. Nevertheless, numerous safe treatment options with minimal complications, for example, topical retinoids, exist for SOC individuals and warrant further exploration. In summary, there is substantial room for improvement in this area, and there is considerable optimism for enhancing intervention strategies in the future.

## Supplemental Material

sj-docx-1-cms-10.1177_12034754241265716 – Supplemental material for Treatment of Post-Inflammatory Hyperpigmentation in Skin of Colour: A Systematic Review Supplemental material, sj-docx-1-cms-10.1177_12034754241265716 for Treatment of Post-Inflammatory Hyperpigmentation in Skin of Colour: A Systematic Review by Kristie Mar, Bushra Khalid, Mahan Maazi, Rayan Ahmed, Ou Jia (Emilie) Wang and Touraj Khosravi-Hafshejani in Journal of Cutaneous Medicine and Surgery

sj-pdf-2-cms-10.1177_12034754241265716 – Supplemental material for Treatment of Post-Inflammatory Hyperpigmentation in Skin of Colour: A Systematic Review Supplemental material, sj-pdf-2-cms-10.1177_12034754241265716 for Treatment of Post-Inflammatory Hyperpigmentation in Skin of Colour: A Systematic Review by Kristie Mar, Bushra Khalid, Mahan Maazi, Rayan Ahmed, Ou Jia (Emilie) Wang and Touraj Khosravi-Hafshejani in Journal of Cutaneous Medicine and Surgery
