# Supplementary material for: Treatment of Post-Inflammatory Hyperpigmentation in Skin of Colour: A Systematic Review
Source: J Cutan Med Surg. 2024 Jul 29;28(5):473–80. doi: 10.1177/12034754241265716 (PMC11514325; doi:10.1177/12034754241265716)
Supplement: sj-docx-1-cms-10.1177_12034754241265716 – Supplemental material for Treatment of Post-Inflammatory Hyperpigmentation in Skin of Colour: A Systematic Review [file sj-docx-1-cms-10.1177_12034754241265716.docx]

**Table S1.** Included studies

| **Country (%)** | United States | 31.1 |
| --- | --- | --- |
|  | Korea | 11.1 |
|  | United Kingdom | 8.9 |
|  | China | 8.9 |
|  | India | 6.7 |
|  | South Africa | 4.4 |
|  | Turkey | 4.4 |
|  | Thailand | 4.4 |
|  | Philippines | 2.2 |
|  | Saudi Arabia | 2.2 |
|  | Netherlands | 2.2 |
|  | Brazil | 2.2 |
|  | Malaysia | 2.2 |
|  | Germany | 2.2 |
|  | France | 2.2 |
|  | Hong Kong | 2.2 |
|  | Mexico | 2.2 |
|  | **Total (n)** | 46 |
| **Study type (%)** | Case report | 31.1 |
|  | RCT | 26.7 |
|  | Experimental | 30.0 |
|  | Prospective cohort | 6.7 |
|  | Retrospective cohort | 6.7 |
|  | Case series | 4.4 |
|  | Observational | 2.2 |
|  | Non-RCT | 2.2 |
|  | **Total (n)** | 46 |
| RCT: Randomized controlled trial; Non-RCT: Non-randomized controlled trial | | |

**Table S2.** SOC patient characteristics and clinical features of PIH

|  | |  | |  | **SOC patients** |
| --- | --- | --- | --- | --- | --- |
| **Total number of patients (n)** | |  | |  | 1356 |
|  | |  | |  |  |
| **Sex (%)** | Females | |  | | 77.7 |
|  | Males | |  | | 22.3 |
|  | **Total (n)** | |  | | 1011 |
| **Pooled mean age (years)** |  | |  | | 29.3 |
|  | **Total (n)** | |  | | 1036 |
| **Ethnicity/Race (%)** | Black | |  | | 69.7 |
|  | Asian | |  | | 27.1 |
|  | Hispanic/Latin | |  | | 2.9 |
|  | Other | |  | | 0.2 |
|  | **Total (n)** | |  | | 849 |
| **Fitzpatrick Skin type (%)** | III | |  | | 20.2 |
|  | IV | |  | | 40.4 |
|  | V | |  | | 33.9 |
|  | VI | |  | | 5.6 |
|  | **Total (n)** | |  | | 570 |
| **Colour of PIH (%)** | Light brown | |  | | 60.0 |
|  | Dark brown | |  | | 33.3 |
|  | Black | |  | | 6.7 |
|  | **Total (n)** | |  | | 15 |
| **Morphology (%)** | Macule | |  | | 10.0 |
|  | Patch | |  | | 90.0 |
|  | **Total (n)** | |  | | 10 |
| **Location (%)** | Face | |  | | 91.8 |
|  | Extremities | |  | | 2.5 |
|  | Axillae | |  | | 4.1 |
|  | Trunk | |  | | 0.1 |
|  | Hands/Feet | |  | | 0.1 |
|  | **Total (n)** | |  | | 1030 |
| **Distribution (%)** | Localized | |  | | 47.5 |
|  | Diffuse | |  | | 45.0 |
|  | Symmetrical | |  | | 7.5 |
|  | **Total (n)** | |  | | 80 |
| **Precipitating factor (%)** | *Inflammatory* | |  | |  |
|  |  | | Acne | | 97.3 |
|  |  | | Photodamage | | 2.5 |
|  |  | | Lichen planus | | 0.2 |
|  |  | | Scleroderma | | 0.1 |
|  |  | | Atopic dermatitis | | 0.1 |
|  |  | | Scleroderma | | 0.1 |
|  | **Total (n)** | |  | | 1089 |
|  | *Trauma* | |  | |  |
|  |  | | Laser therapy | | 26.9 |
|  |  | | Hair removal | | 26.1 |
|  |  | | Light therapy | | 23.1 |
|  |  | | Other physical trauma | | 2.0 |
|  |  | | Chemical peel | | 20.1 |
|  |  | | Cupping | | 0.7 |
|  |  | | Aesthetic plasma exeresis | | 0.7 |
|  | **Total (n)** | |  | | 134 |
|  | *Medication* | |  | |  |
|  |  | | Unidentified drug eruption | | 100.0 |
|  | **Total (n)** | |  | | 2 |
| **Length of symptoms prior to treatment (months)** |  | |  | | 20.8 |
|  | **Total (n)** | |  | | 167 |

**Table S3.** Treatments and associated outcomes for SOC patients

| **Treatment** | | | | **Sample (%)** | **Mean number of treatments** | | **Complete response (%)** | **Partially reduced pigment (%)** | **Partially reduced size (%)** | **No response (%)** | **Resolution time (days)** | **Follow up (months)** |
| --- | --- | --- | --- | --- | --- | --- | --- | --- | --- | --- | --- | --- |
|  | | |  |  |  | |  |  |  |  |  |  |
|  | No treatment | | | 25.3 | 100.8 | | 0 (0/117) | 62.4 (73/117) | 4.3 (7/117) | 33.3 (39/117) | 68.0 | 2.6 |
|  | **Total (n)** | | | 346 |  | |  |  |  |  | 55 | 130 |
| *Monotherapeutic topical regimen (34.4%)* | | | | |  | |  |  |  |  |  |  |
|  | | | |  |  | |  |  |  |  |  |  |
|  | Chemical peels | | | 9.3 | 4.7 | | 0 (0/60) | 66.7 (40/60) | 0 (0/60) | 33.3 (20/60) | 28.0 | 3.9 |
|  | **Total (n)** | | | 123 |  | |  |  |  |  | 8 | 70 |
|  |  | | |  |  | |  |  |  |  |  |  |
|  | Topical retinoids | | | 22.2 | 119.1 | | 0 (0/118) | 64.4 (76/118) | 21.2 (25/118) | 14.4 (17/118) | - | 3.8 |
|  | **Total (n)** | | | 294 |  | |  |  |  |  |  | 143 |
|  |  | | |  |  | |  |  |  |  |  |  |
|  | Topical Bakuchiol | | | 1.5 | 56 | | - | - | - | - | - | 1.0 |
|  | **Total (n)** | | | 20 | 20 | |  |  |  |  |  | 18 |
|  |  | | |  |  | |  |  |  |  |  |  |
|  | Topical niacinamide | | | 1.2 | 63.0 | | 0 (0/16) | 68.8 (11/16) | 0 (0/16) | 31.3 (5/16) | NR | 2.25 |
|  | **Total (n)** | | | 16 |  | |  |  |  |  |  | 16 |
|  |  | | |  |  | |  |  |  |  |  |  |
|  | Topical cysteamine | | | 0.1 | - | | 100.0 (1/1) | 0 (0/1) | 0 (0/1) | 0 (0/1) | 112.0 | NR |
|  | **Total (n)** | | | 1 |  | |  |  |  |  | 1 |  |
|  |  | | |  |  | |  |  |  |  |  |  |
|  | Lumixyl, SilkPeel (mechanical exfoliation) | | | 0.1 | 80.0 | | - | - | - | - | - | - |
|  | **Total (n)** | | | 1 |  | |  |  |  |  |  |  |
|  | | |  |  |  | |  |  |  |  |  |  |
| *Combination topical regimen (12.8%)* | | | |  |  | |  |  |  |  |  |  |
|  | | |  |  |  | |  |  |  |  |  |  |
|  | Topical hydroquinone, topical retinoids, other | | | 6.1 | 46.9 | | 0 (0/41) | 92.7 (38/41) | 0 (0/41) | 7.3 (3/41) | 96.0 | 3.9 |
|  | **Total (n)** | | | 81 |  | |  |  |  |  | 19 | 80 |
|  |  | | |  |  | |  |  |  |  |  |  |
|  | Topical Thiamidol, sunscreen | | | 4.8 | 168 | | 0 (0/64) | 100.0 (64/64) | 0 (0/64) | 0 (0/64) | - | - |
|  | **Total (n)** | | | 64 |  | |  |  |  |  |  |  |
|  |  | | |  |  | |  |  |  |  |  |  |
|  | Topical antibiotics, retinoids | | | 1.1 | - | | 0 (0/10) | 50.0 (5/10) | 50.0 (5/10) | 0 (0/10) | 84.0 | 3.0 |
|  | **Total (n)** | | | 15 |  | |  |  |  |  | 10 | 10 |
|  |  | | |  |  | |  |  |  |  |  |  |
|  | Topical hydroquinone, topical steroids, other | | | 0.7 | 1.7 | | 0 (0/8) | 37.5 (3/8) | 50.0 (4/8) | 12.5 (1/8) | 252.0 | 1.5 |
|  | **Total (n)** | | | 9 |  | |  |  |  |  | 1 | 9 |
|  |  | | |  |  | |  |  |  |  |  |  |
|  | Topical steroids, other | | | 0.1 | - | | 100.0 (1/1) | 0 (/1) | 0 (0/1) | 0 (0/1) | - | - |
|  | **Total (n)** | | | 1 |  | |  |  |  |  |  |  |
|  | |  | |  |  | |  |  |  |  |  |  |
| *Monotherapeutic systemic regimen (0.2%)* | | | | |  | |  |  |  |  |  |  |
|  | | | |  |  | |  |  |  |  |  |  |
|  | Systemic retinoids | | | 0.2 | 168.0 | | 0 (0/2) | 50.0 (1/2) | 50.0 (1/2) | 0 (0/2) | - | 4.0 |
|  | **Total (n)** | | | 2 |  | |  |  |  |  |  | 1 |
|  | |  | |  |  | |  |  |  |  |  |  |
| *Monotherapeutic device regimen (25.0%)* | | | | |  | |  |  |  |  |  |  |
|  | | | |  |  | |  |  |  |  |  |  |
|  | Laser therapy | | | 17.1 | 3.6 | | 26.1 (43/165) | 66.1 (109/165) | 4.2 (7/165) | 3.6 (6/165) | 139.5 | 7.9 |
|  | **Total (n)** | | | 227 |  | |  |  |  |  | 22 | 141 |
|  |  | | |  |  | |  |  |  |  |  |  |
|  | IPL | | | 6.4 | 2.4 | | 1.2 (1/85) | 55.3 (47/85) | 30.6 (26/85) | 12.9 (11/85) | 105.0 | 5.6 |
|  | **Total (n)** | | | 85 |  | |  |  |  |  | 23 | 85 |
|  |  | | |  |  | |  |  |  |  |  |  |
|  | High-intensity focused ultrasound | | | 1.5 | 1.0 | | 0 (0/8) | 100.0 (8/8) | 0 (0/8) | 0 (0/8) | 28.0 | 1.0 |
|  | **Total (n)** | | | 20 |  | |  |  |  |  | 8 | 8 |
|  |  | | |  |  | |  |  |  |  |  |  |
| *Combination device and topical regimen (1.4%)* | | | | |  | |  |  |  |  |  |  |
|  | | | |  |  | |  |  |  |  |  |  |
|  | Topical hydroquinone, laser therapy | | | 1.4 | - | | 0 (0/19) | 100.0 (19/19) | 0 (0/19) | 0 (0/19) | - | - |
|  | **Total (n)** | | | 19 |  | |  |  |  |  |  |  |
|  | |  | |  | |  |  |  |  |  |  |  |
| IPL: intense pulsed light; IVIG: Intravenous immunoglobulins; TXA: Tranexamic acid | | | | | | | | | | | | |
