# Supplementary material for: Treatment of Post-Inflammatory Hyperpigmentation in Skin of Colour: A Systematic Review
Source: J Cutan Med Surg. 2024 Jul 29;28(5):473–80. doi: 10.1177/12034754241265716 (PMC11514325; doi:10.1177/12034754241265716)
Supplement: sj-pdf-2-cms-10.1177_12034754241265716 – Supplemental material for Treatment of Post-Inflammatory Hyperpigmentation in Skin of Colour: A Systematic Review [file sj-pdf-2-cms-10.1177_12034754241265716.pdf]

**Figure S1.** PRISMA flow diagram

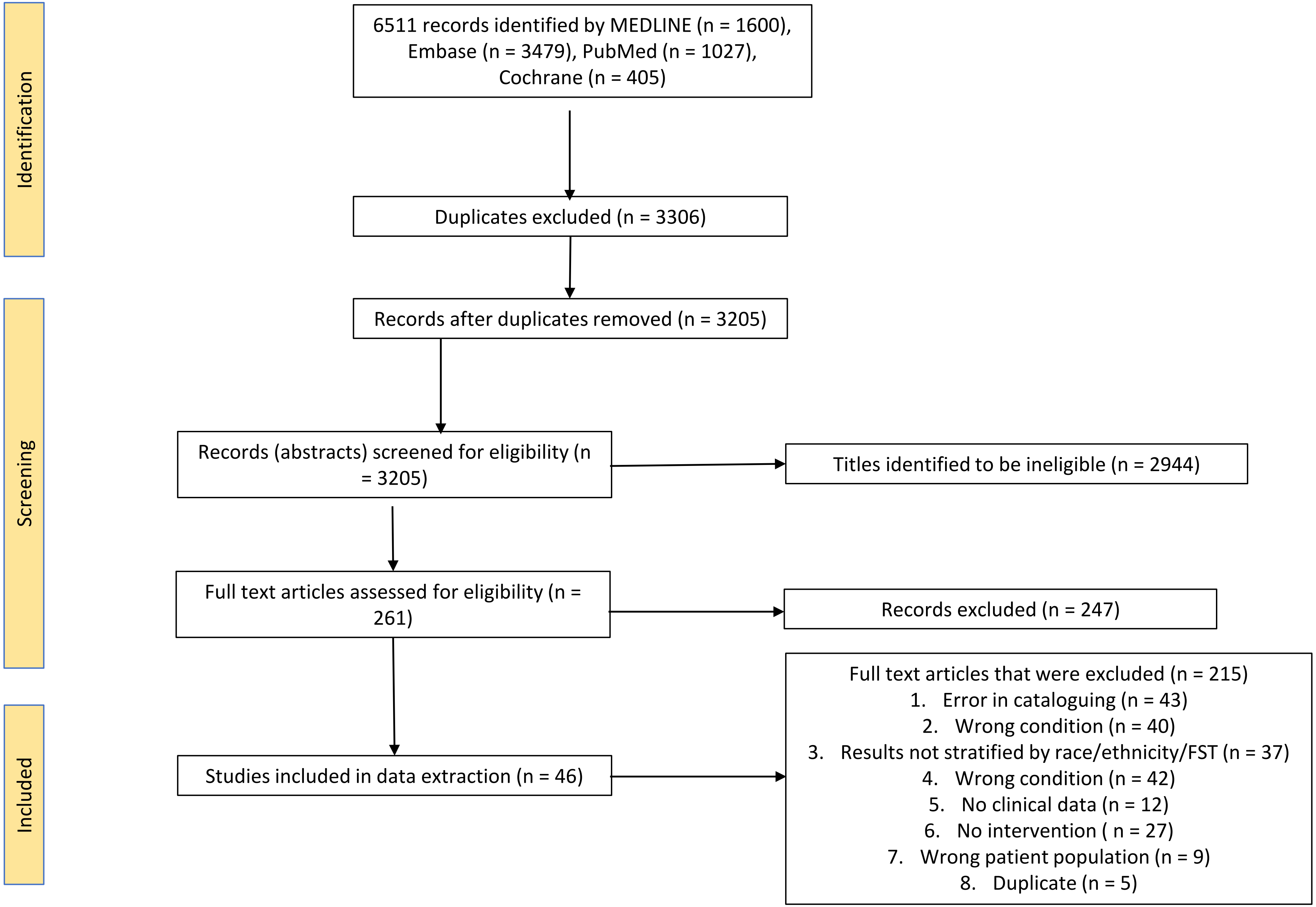

# Figure S2

| **Supplementary Figure 1.** Summary of included studies | | | | | | | | | | | | |
|---|---|---|---|---|---|---|---|---|---|---|---|---|
| **Author, Year, Country** | **Sample size (n), Sex (M:F)** | **Mean Age, (Age range)** | **Race / Ethnicity (n), Fitzpatrick** | **Morphology (n) / Location (n)** | **Duration of PIH (days)** | **Precipitating Factor (n)** | **Treatment(s) / Prevention(s)** | **Intervention parameters** | **Outcomes** | **Follow up in months (n)** | **Recurrence** | **Adverse Events** |
| Grayson & Heath, 2020, USA | 1 (1:0) | 53 | Black (1), NR | Patch (1) / Trunk (1) Extremities (1) Face (1) | 365 | Atopic dermatitis (1) | Triamcinolone 0.1%, tacrolimus 1%, cetirizine 20 mg and dupilumab 600mg loading and 200mg maintenance (1) | Cetirizine PO daily, dupilumab loading dose and qbi-weekly | Complete response (1) | NR | 1 | NR |
| Robredo, 2020, USA | 9 (0:9) | 39 (20-52) | Asian (9), NR | NR / Axillae (18) | NR | NR | 1064-nm QSNY laser therapy (9) | qbi-weekly for four sessions | Reduced pigment (9) | 3 (9) | 0 | None |
| Roggenkamp et al., 2021, Germany | 93 (NR) | NR (18-50) | NR | NR / Face (88) | NR | Acne (93) | Thiamidol, oil in water (64) | qdaily for 168 days | Reduced pigment (64) | NR | NR | None |
| Mathe et al., 2020, UK | 1 (0:1) | 20 | NR, V (1) | Patch (1) / Face (1) | 7200 | Scleroderma morphea (1) | Cysteamine (1) | qdaily | Reduced pigment (1) | NR | NR | NR |
| How et al., 2020, Malaysia | 36 (13:23) | NR | NR, III (2) IV (20) V (14) | NR / Face (36) | NR | Acne (36) | JP (14g resorcinol, 14g SA, 14mL of lactic acid, ethanol quantum satis 100mL) chemical peel (34) | 3 sessions | Reduced pigment (26), no response (8) | NR | NR | Erythema (4), acneiform eruption (6), burning and stinging (NR) |
| | | | | | | | SA (34) | 3 sessions | Reduced pigment (29), no response (5) | NR | NR | Erythema (5) |
| Nipshagen et al., 2020, Netherlands | 1 (0:1) | 61 | NR, IV (1) | Light brown, symmmetrical / Face (1) | 1.28 | Aesthetic plasma exeresis (1) | Hydroquinone, tretinoin and thermomechanical ablation (1) | NR | Reduced pigment (1) | NR | 0 | NR |
| Ren & Zhao, 2022, China | 1 (0:1) | 36 | Asian (1), NR | Localized light brown macules, Face (1) | 730 | NR | 755-nm alexandrite picosecond laser combined with a FOCUSTM diffractive lens array (1) | 2 sessions | Complete response (1) | 24 (1) | 0 | NR |
| Wang et al., 2022, China | 60 (8:52) | 29 (22-37) | NR, III (38) IV (22) | NR | 36 | Acne | 400-1200-nm IPL (60) | Range of 2-7 sessions | Complete response (1), reduced pigment (22), decreased size (26), no response (11) | 6 (60) | 7 | Transient erythema, dryness, itching (NR) |
| Ren et al., 2021, China | 1 (1:0) | 38 | Asian (1), III (1) | Localized light brown patch / Face (1) | 365 | Other physical trauma (1) | 755-nm alexandrite picosecond laser combined with a FOCUSTM diffractive lens array (1) | 3 sessions | Reduced pigment (1) | 36 (1) | 0 | Deepening of skin lesion (1) |

**Supplementary Figure 1.** Summary of included studies

| **Author, Year, Country** | **Sample size (n), Sex (M:F)** | **Mean Age, (Age range)** | **Race / Ethnicity (n), Fitzpatrick** | **Morphology (n) / Location (n)** | **Duration of PIH (days)** | **Precipitating Factor (n)** | **Treatment(s) / Prevention(s)** | **Intervention parameters** | **Outcomes** | **Follow up in months (n)** | **Recurrence** | **Adverse Events** |
|---|---|---|---|---|---|---|---|---|---|---|---|---|
| Alharbi, 2021, Saudi Arabia | 9 (0:9) | 30 (18-44) | NR, III (9) | NR / Trunk (4) Extremities (2) Hands/feet (1) Face (2) | 203.792 | Acne (1), chemical peel (1), atopic dermatitis (1), laser (2), postmammoplasty (1), postliposuction (1), burn (1) postabdominoplasty (1) | Laser, oral steroid, clobetasol cream, topical hydroquinone (9) | 1 session | Reduced pigment (3), decreased size (4), no response (1) | 1.5 (9) | NR | NR |
| Feng & Huang, 2021, China | 15 (NR) | 34 (24-51) | NR, III (3) IV (12) | NR / Face (15) | 1387.95 | Acne (5), IPL (2), drug eruption (2), idiopathic melanosis (1), cat scratch (1), allergy (1), thermal damage (3) | 1064-nm QSNY laser (15) | NR | Complete response (4), reduced pigment (5), decrased size (3), no response (3) | NR | NR | Mild erythema (NR) |
| Bhatia et al., 2020, USA | 308 (99:209) | 22 (NR) | NR | NR / Face (308) | NR | Acne (308) | Topical tretinoin (165) | qdaily for 120 days | NR | NR | NR | Pain (4), dryness (4), erythema (2), exfoliation (2), irritation (2) |
| | | | | | | | Control (143) | qdaily for 120 days | NR | NR | NR | Pain (1), erythema (1) |
| Lyons et al., 2020, USA | 20 (6:14) | NR | NR | Localized / Extremities (20), face (20) | | Acne (20), trichloroacetic acid (20) | Bakuchiol or vehicle cream (20) | NR | NR | 1 (20) | NR | NR |
| Bae et al. 2020, USA | 61 (5:56) | 46.5 (29-72) | NR / III (0) IC (45) V (10) VI (6) | NR / Hands/feet (8) face (42) | NR | NR | Low energy fractionated 1927-nm laser (61) | Range from 2-5+ sessions | NR | NR | NR | None |
| Lueangarun et al., 2019, Thailand | 20 (12:8) | 38 (26-46) | NR / III (6) IV (11) V (2) | NR / Face (20) | NR | Laser (20) | Topical hydroquinone, tretinoin, steroids and others cream (20) | BID for 7 days | NR | 2 (20) | NR | Acneiform eruption (1) |
| | | | | | | | Control (20) | BID for 7 days | NR | 2 (20) | NR | Acneiform eruption (1) |
| Sakar et al., 2019, India | 45 (15:30) | 23.2 (16-38) | NR | NR / Face (40) | 1163 | Acne (45) | GA (15), SA (15), phytic acid (15) chemical peels | qbi-weekly for 6 sessions | NR | 3 (45) | NR | Burning (4), erythema (1) |
| Wilson et al., 2018, USA | 40 (4:36) | 46.6 (29-71) | NR / III (20) IV (14) V (6) | NR / Face (40) | NR | Photodamage (27) | Laser treatment, hydroquinone cream (19) | 4 sessions of laser with 168 qdaily days of HQ | Reduced pigment (19) | 3 (19) | NR | None |
| | | | | | | | Nonablative, fractional, 1927-nm diode laser, regular face cream (19) | 4 sessions of laser with 168 qdaily days of cream | Reduced pigment (19) | 3 (19) | NR | None |

**Supplementary Figure 1.** Summary of included studies

| Author, Year, Country | Sample size (n), Sex (M:F) | Mean Age, (Age range) | Race / Ethnicity (n), Fitzpatrick | Morphology (n) / Location (n) | Duration of PIH (days) | Precipitating Factor (n) | Treatment(s) / Prevention(s) | Intervention parameters | Outcomes | Follow up in months (n) | Recurrence | Adverse Events |
|---|---|---|---|---|---|---|---|---|---|---|---|---|
| Sarkar et al., 2017, India | 30 (4:26) | 30 (21-40) | Asian (30) / NR | NR / Face (30) | NR | NR | MKF, GA chemical peels (15) | q3weeks for 6 sessions | Reduced pigment (13), no response (2) | NR | NR | Erythema, burning, desquamation (8) vesiculation (3) |
| | | | | | | | MKF (15) | qdaily for 126 days | Reduced pigment (12), no response (3) | 5.3 (15) | NR | Erythema (5) residual hyperpigmentation (3) |
| Park et al., 2016, Korea | 25 (0:25) | 40 (25-58) | Asian (25) / III (3) IV (20) V (2) | NR / Face (25) | NR | Acne (1) laser (16) atopic dermatitis (6) photodamage (2) | Pulse-in-pulse mode IPL (25) | qweekly for 4 sessions, qbi-weekly for 4 sessions | Reduced pigment (25) | 4.8 (25) | NR | None |
| Campos & Favaro, 2016, Brazil | 41 (NR) | 28 (18-35) | NR / III (9) IV (20) V (12) | Localized (8) diffuse (34) / Face (41) | NR | Acne (41) | Topical dioic acid, salicylic acid, glycolid acid, LHA, citric acid, and HEPES (42) | qdaily for 56 days | NR | 1.9 (41) | NR | None |
| Zaware et al., 2015, India | 78 (13:65) | 23 (18-27) | Asian (78) / NR | NR / Face (78) | NR | Acne (73) | 1064-nm QSNY laser therapy (73) | qbi-weekly for 6 sessions | Complete response (32) reduced pigment (41) | 3 (73) | NR | Erythema, edema, and punctate bleeding, mild crusting (NR) |
| Colvan et al., 2015,USA | NR | NR (23-37) | NR | NR / Face (8) | NR | Acne (8) | Chemical peel (lactic acid, salicylic acid, resorcinol and retinol), topical acne lotion (8) | qmonthly for 3 peels and qdaily for 168 days of lotion | NR | NR | NR | NR |
| Oram & Akkaya, 2014, Turkey | 1 (0:1) | 24 | NR / III (1) | Localized light brown patch (1) / Face (1) | 2190 | Hair removal (1) | Fractional CO2 laser laser therapy (1) | 2 sessions qmonthly | Complete response (1) | NR | NR | NR |
| Lee et al., 2014, Korea | 1 (0:1) | 26 | Asian (1) / IV (1) | Localized light brown patch (1) / Trunk (1) | 180 | Cupping (1) | 1927-nm thulium fiber laser therapy (1) | 4 sessions qmonthly | Reduced pigment, decrased size (1) | 4 (1) | 0 | Mild pain, moderate postprocedural erythema and edema (1) |
| Winhoven et al., 2005, UK | 1 (0:1) | 23 | Asian (1) / NR | Diffuse dark brown patch / Face (1) | NR | Acne (1) | Oral isotretinoin 60mg (1) | qdaily | Complete response (1) | 4 (1) | NR | NR |
| Jacyk, 2001, South Africa | 44 (NR) | NR (12-30) | Black (44) / NR | NR / Face (44) | NR | Acne (44) | Topical adapalene (44) | qdaily for 84 days | NR | 3 (44) | NR | None |
| Burns et al., 1997, USA | 19 (0:19) | NR (25-63) | Black (19) / NR | NR / Face (19) | NR | NR | GA chemical peels (2%HQ/10%GA, 0.05% tretinion) (10) | q3weeks for 6 sessions | NR | 5.5 (9) | NR | Mild erythema, superficial desquamation, superficial vesiculation (NR) |
| | | | | | | NR | HQ/GA/tretinoin only (9) | BID | NR | 5.5 (7) | NR | NR |
| Takiwaki et al., 1994, Japan | 16 (16:0) | 28 (24-38) | Asian (16) / III (16) | NR / Trunk (16) | NR | NR | Topical clobetasol (16) | 1 session | NR | NR | NR | NR |
| Taylor & Anderson, 1994, USA | 8 (2:2) | 39 (27-65) | NR / III (1) VI (2) | NR / Trunk (1) extremities (3) | NR | Lichen planus (2), physical trauma (1) | Laser therapy (8) | NR | No response (3) | NR | NR | Erosions (2), worsening PIH (3), patchy hypopigmentation (NR), hyperpigmentation (NR) |

**Supplementary Figure 1.** Summary of included studies

| Author, Year, Country | Sample size (n), Sex (M:F) | Mean Age, (Age range) | Race / Ethnicity (n), Fitzpatrick | Morphology (n) / Location (n) | Duration of PIH (days) | Precipitating Factor (n) | Treatment(s) / Prevention(s) | Intervention parameters | Outcomes | Follow up in months (n) | Recurrence | Adverse Events |
|---|---|---|---|---|---|---|---|---|---|---|---|---|
| Bulengo-Ransby, 1993, USA | 33 (9:24) | 33 (9-24) | Black (33) / NR | NR | NR | Acne (62) hair removal (10) | Topical tretinoin (24) | qdaily for 280 days | Reduced pigment (22) | NR | NR | Retinoid dermatitis (12) |
| | 35 (12:23) | 34 (20-65) | NR | NR | NR | NR | Control (30) | qdaily for 280 days | Reduced pigment (17) no response (13) | NR | NR | NR |
| Pearson et al., 2023, USA | 1 (0:1) | 32 | Black (1) | Dark brown, black diffuse irregular (1) / Face (1) | NR | Physical trauma (1) | Topical steroids (1) | NR | No response (1) | 5 (1) | NR | NR |
| Lee et al., 2017, Korea | 1 (0:1) | 20 | Asian (1) / IV (1) | Localized light brown patch (1) / Face (1) | NR | NR | 750-picosecond pulse, 755-nm Alexandrite laser therapy (1) | qbi-weekly for 7 sessions | Reduced pigment, decreased size (1) | NR (0) | NR | None |
| Bhatia et al., 2014, USA | 1 (NR) | NR | NR / IV (1) | NR | NR | NR | Other tyrosinase inhibitor (Lumixyl) (1) | BID for 40 days | NR | NR | NR | NR |
| Callender et al., 2012, USA | 15 (NR) | NR | Black (15) / NR | NR | NR | Acne (15) | Topical clindamycin, tretinoin (15) | BID for 84 days | Reduced pigment (5) decreased size (5) | 3 (10) | NR | Minimal scaling, itching, burning (NR) |
| | 15 (NR) | NR | Black (15) / NR | NR | NR | Acne (15) | Control (15) | BID for 84 days | Reduced pigment (5) decreased size (5) | 3 (10) | NR | Minimal scaling, itching, burning (NR) |
| Rossi et al., 2011, France | 25 (NR) | NR | NR | NR | NR | Acne (25) | Topical tretinoin (25) | NR | Reduced pigment, decrased size (25) | 3 (25) | NR | NR |
| Ho et al., 2010, Korea | 34 (0:34) | 35 (21-49) | Asian (34) / III (11) IV (20) V(3) | NR | NR | Acne (34) | Topical HQ, tretinoin, steroids, and others cream (8) | 66 applications | Reduced pigment (8) | 6.3 (8) | NR | NR |
| | | | | | | | Topical HQ, tret, steroids, others cream and laser therapy (17) | Topical for 6.9 weeks and laser 5 sessions | Reduced pigment (17) | 5.9 (17) | NR | Hyperpigmentation (1) |
| | | | | | | | 595-nm long pulsed dye laser and/or 1064-nm QSNY laser therapy (9) | 5 sessions | Reduced pigment (9) | 6 (9) | NR | NR |
| Kim & Cho, 2010, Korea | 5 (3:2) | 35 (30-42) | Asian (5) / NR | Localized (4), symmterical (1), light brown (3), dark brown (2), patch (1), irregular (4) / Face (5) | 210 | IPL (2), laser (2), chemical peels (2) | 1064-nm QSNY laser therapy (5) | qweekly for 5 sessions | Reduced pigment (5) | 126 (5) | 0 | NR |

| **Supplementary Figure 1.** Summary of included studies | | | | | | | | | | | | |
|---|---|---|---|---|---|---|---|---|---|---|---|---|
| **Author, Year, Country** | **Sample size (n), Sex (M:F)** | **Mean Age, (Age range)** | **Race / Ethnicity (n), Fitzpatrick** | **Morphology (n) / Location (n)** | **Duration of PIH (days)** | **Precipitating Factor (n)** | **Treatment(s) / Prevention(s)** | **Intervention parameters** | **Outcomes** | **Follow up in months (n)** | **Recurrence** | **Adverse Events** |
| Kim & Cho, 2010, Korea | 40 (NR) | 23 (19-27) | Asian (40) / NR | NR | NR | Acne (40) | 1064-nm QSNY laser therapy (20) | qweekly for 5 sessions | Complete response (4) reduced pigment (16) | 3 (20) | NR | Mild to moderate erythema (NR) |
| | | | | | | | Acne extractions (20) | qweekly for 5 sessions | Reduced pigment (15), no response (5) | 3 (20) | NR | Mild to moderate erythema (NR) |
| Cho et al., 2009, Korea | 3 (1:2) | 43 (31-58) | Asian (3) / NR | Diffuse light brown patch (3) / Face (3) | 110 | Laser (3) | 1064-nm QSNY laser therapy (3) | qweekly for 5 sessions | Complete response (1), reduced pigment (2), decreased size (2) | 2 (3) | 0 | None |
| Joshi et al., 2009, USA | 10 (0:10) | 33 (23-49) | Black (8) Asian (2) / IV (2) V (6) VI (2) | NR | NR | NR | SA chemical peels (10) | qbi-weekly for 5 sessions | No response (10) | NR | NR | Burning (1) redness (4) dryness (1) crusting (4) itching (7) hyperpigmentaiton (4) hypopigmentation (1) |
| | | | | | | | Control (10) | NA | No response (10) | NR | NR | NR |
| Alexis & Lamb, 2009, UK | 1 (1:0) | NR | NR | Symmetrical, localized (1) / Face (1) | 1095 | Acne (1) | SA chemical peels (1) | q6weeks for 4 sessions | Reduced pigment (1) | 10.5 (1) | 0 | NR |
| Grimes & Callender, 2007, USA | 74 (9:65) | 35 (12-84) | Black (69) Asian (1) Hispanic (1) Other (2) / III (2) IV (14) V (41) VI (17) | NR / Face (74) | NR | Acne (74) | Topical tazarotene cream (36) | qdaily for 126 days | NR | 4.5 (74) | NR | None |
| | | | | | | | Control (38) | qdaily for 126 days | NR | NR | NR | NR |
| Winhoven et al., 2005, UK | 1 (0:1) | 23 | Asian (1) / NR | Symmetrical dark brown patch (1) / Face (1) | 3650 | Acne (1) | Systemic isotretinoin (1) | NR | Reduced pigment, decreased size (1) | 4 (1) | NR | NR |
| Jacyk, 2001, South Africa | 44 (NR) | NR (12-30) | Black (44) / NR | NR | NR | Acne (44) | Topical tretinoin (44) | qdaily for 90 days | Reduced pigment (29), no response (15) | 3 (44) | NR | NR |
| Castanedo-Cazares et al., 2012, Mexico | 24 (0:24) | 21 (19-27) | Middle Eastern (24) / III (5) IV (11) V (8) | Axillae (24) | 139 | Hair removal (24) | Topical niacinamide (16) | qdaily for 63 days | Reduced pigment (11) no response (5) | 2.3 (16) | NR | NR |
| | | | | | | | Topical desonide (16) | qdaily for 63 days | Reduced pigment (10) no response (6) | 2.3 (16) | NR | NR |
| | | | | | | | Control (16) | qdaily for 63 days | Reduced pigment (6) no response (10) | 2.3 (16) | NR | NR |

HEPES: 4-(2-hydroxyethyl)-1-piperazineethanesulfonic acid; IPL: intense pulsed light; LHA: Lipo Hydroxy Acid; EGF: Epidermal growth factor; QSNY: Q-switched neodymium-doped yttrium aluminum garnet; CO2: Carbon dioxide; SA: Salicylic acid; GA: Glycolic acid; JS: Jessner's solution; MKF: Modified Kligman's formula (hydroquinone 2%, tretinoin 0.05%, hydrocortisone 1%); NR: Not reported
